# Methanogenic community during the anaerobic digestion of different substrates and organic loading rates

**DOI:** 10.1002/mbo3.709

**Published:** 2018-08-15

**Authors:** Dewang Kong, Keqiang Zhang, Junfeng Liang, Wenxuan Gao, Lianzhu Du

**Affiliations:** ^1^ Agro‐Environmental Protection Institute Ministry of Agriculture Tianjin China; ^2^ College of Land and Environment Shenyang Agricultural University Shenyang China

**Keywords:** anaerobic digestion, maize straw, methanogenic community, organic loading rate, pig manure

## Abstract

Three anaerobic reactors using pig manure (PM), maize straw (MS), and a mixture of the two as substrates were compared for archaeal community structure and diversity, and for methanogens response to increased organic loading rate (OLR, expressed in the mass of volatile solid (VS)). Methanogenic archaeal richness during codigestion of pig manure with maize straw (ACE: 2412) was greater than that during the others (ACE: 1225, 1467) at an OLR of 4 g L^−1^ day^−1^, accompanied by high specific methane yield. *Euryarchaeota* and *Crenarchaeota* predominated during overall digestion of different substrates; with relative abundances of 63.5%–99.0% and 1.0%–36.3%, respectively. *Methanosarcina* was the predominant genus that accounted for 33.7%–79.8% of the archaeal community. The diversity in the PM digester decreased with increase in OLR, but increased in the MS digester. The diversity was stable during the codigestion with increased OLR. The relative abundances of hydrogenotrophic methanogens increased by 2.6 and 2.1 folds; the methanogenic community shifted from acetoclastic to hydrogenotrophic methanogens during digestion of MS, and of the mixture of MS and PM. Canonical correspondence analysis revealed a strong relationship between reactor parameters and methanogenic community.

## 1 INTRODUCTION

China is a large agricultural country in which abundant biomass resources are generated during agricultural processes. In 2012, 846 million tons of crop residues and 3.21 billion tons of livestock manure were produced; had these wastes been utilized for anaerobic fermentation, their biogas potential would have been 4.23 × 10^11^ m^3^ (Li, Liu, & Sun, 2016). The production of livestock manure was around 3.49 billion tons in 2016 (National Bureau of Statistics of the People's Republic of China, 2017; Zhang, Bo, & Geng, 2012). It was reported that the total biogas production from these agricultural wastes was 2 billion m^3^, implying that less than 5% of the waste was utilized for biogas production in 2014 (Chen, Cong, Shu, & Mi, 2017; Zhang, Yang, & Xie, 2015). Worldwide, large amounts of livestock and poultry manure have become concentrated in certain areas over the last few decades, as agricultural land is not sufficient for their recycling via the soil‐plant system. This fact together with the inadequate management of these wastes, has caused serious land, water, and air pollution problems (Chen & Liu, 2017; Li, Liu, et al., 2016) that have received increased attention from environmentalists, economists, and policymakers (Li, Cheng, Yu, & Yang, 2016).

Anaerobic digestion (AD), also called biogas fermentation, is an important microbial process for the generation of renewable energy and reduction in environmental pollution; it promotes ecofriendly agricultural land by the application of digester residues simultaneously (Wang et al., 2016). In addition, AD has significant advantages over other forms of waste treatment, such as less biomass sludge, minimal odor emissions, and so on (Smet, Van Langenhove, & De Bo, 1999; Ward, Hobbs, Holliman, & Jonesw, 2008). The characteristics of agricultural waste, such as imbalance in nutrition and high proportion of proteins or lignocellulosic biomass pose a challenge for process engineering. Furthermore, they affect the microbial community involved in AD, since the high concentration of ammonia likely inhibits methanogens activity, and high fiber content can cause blockage of pumps or sinking or floating layers (Munk, Guebitz, & Lebuhn, 2017).

AD is a complicated microbial process which involves four sequential steps: hydrolysis, acidogenesis, acetogenesis, and methanogenesis. Its performance and stability is strongly related with the microbial community structure. Archaea, especially methanogens, are key players during methanogenesis, thus attracting much attention of researchers (Li, Rui, et al., 2015). Methane is produced through hydrogen oxidation and acetate cleavage by hydrogenotrophic and acetoclastic methanogens, respectively (Kim et al., 2014). Munk et al. (2017) evaluated the potential utility of grass silage, which is rich in nitrogen. The result showed that the reactors could be operated stably as sole substrate at low OLR, although the ammonia concentration was high and hydrogenotrophic methanogens were dominant in the thermophilic and mesophilic reactors. Despite some novel findings, these results were very limited.

Due to the accumulation of inhibitory intermediates such as volatile fatty acids (VFAs) and ammonia, AD of agricultural waste is more prone to failure at high OLR compared to that at a low OLR. Investigation of the structure and dynamics of microbial community during the process of elevating the OLR should enable elucidation of the syntrophic interactions in AD, which may be used to optimize operational conditions. In this study, the typical agricultural wastes like MS, PM, and mixture of MS and PM were digested in laboratory‐scale completely mixed anaerobic reactors, at different OLR of 2 and 4 g L^−1^ day^−1^ for 219 days. Methanogenic community was investigated using high‐throughput sequencing technology. The objective of this study was to compare the structure of methanogenic community in AD with different substrates (PM, MS, and PM+MS), analyze the changes in methanogens due to elevated OLR, and elucidate the link between methanogenic community and reactor performance. It is expected that the results presented herein would enable the optimization of operational conditions in order to achieve high efficiency AD for agricultural applications.

## 2 MATERIALS AND METHODS

### 2.1 Substrates and inoculum

Both PM and MS were collected from Yi Lilai Breeding Co. Ltd. (Xiqing district, Tianjin, China). MS was smashed to fractions of nearly 1 mm in size by laboratory blender (Waring Commercial, USA). PM and MS were stored at 4 ± 1°C. The inoculum sludge was taken from a lab‐scale completely mixed reactor that ran AD of PM, at 35°C. The characteristics of the substrates and inoculum sludge are shown in Table 1.

**Table 1:** The characteristics of PM, MS, and inoculum sludge

	TS (%)	VS (%)	VS/TS (%)	TKN (mg g^−1^ TS)	TOC (mg g^−1^ TS)
PM	30.46 (30.05–31.03)	22.22 (21.62–22.45)	72.34 (71.71–73.52)	35.38 (30.50–42.27)	376.58 (339.12–418.95)
MS	88.50 (88.48–88.52)	80.72 (80.37–80.93)	91.20 (90.79–91.42)	9.85 (9.79–9.90)	431.51 (428.26–434.79)
Inoculum	4.62 (4.60–4.65)	3.79 (3.73–3.83)	82.03 (81.80–82.19)	—	—

***Note.***

‘−’: not detected.

Values are expressed as average (range).

### 2.2 Anaerobic reactor operation

The experiment was carried out in three completely mixed anaerobic digesters with 7 L working volume and mesophilic condition (35 ± 0.5°C). Three reactors were seeded with inoculum sludge (4 L) and adjusted to 7 L with tap water. The substrates were PM (R1), mixture of PM and MS (R2), and MS (R3), respectively. The VS ratio of PM/MS in R2 was 2:1, which maintained the ratio of *C*/*N* at around 26. The substrates were manually added to the reactors after discharging the digestate from the outlet each day. The reactors were run with a start‐up OLR of 2 g L^−1^ day^−1^, this was increased to 3 and 4 g L^−1^ day^−1^ on the 69th and 150th day at corresponding HRTs (60, 40, 30 days).

### 2.3 Physicochemical analysis

Biogas was collected in an aluminum bag (20 L), the volume of which was measured with a wet gas meter every day. Gas and slurry samples were taken from the reactors at intervals of 3 day. Biogas composition was determined by GC (gas chromatography, Thermal Trace‐1300) equipped with TCD (thermal conductivity detector). Concentrations of VFA (acetate, propionate, isobutyrate, butyrate, isovaleric acid, valeric acid) were determined by GC equipped with FID (flame ionization detector) (Zhang, Guo, et al., 2015). Total solids (TS), VS, total kjeldahl nitrogen (TKN), total organic carbon (TOC), alkalinity, and ammonia concentration were analyzed using standard methods (APHA, 1998).

SMY (specific methane yield, ml g^−1^ day^−1^) was the daily methane yield of per gram VS, the value equals daily biogas volume times methane content and divide by VS mass.

### 2.4 DNA extraction and sequencing

Biomass was sampled at the end of each stage, running with OLR of 2 and 4 g L^−1^ day^−1^, respectively, and frozen at −80°C immediately. Total genomic DNA was extracted using Fast DNAs Spin Kit for soil (MP Biomedicals, USA). DNA concentration was checked using a NanoDrop 2000 spectrophotometer (Thermo Scientific, USA). High‐throughput sequencing was conducted using an Illumina MiSeq platform. The primer pair of 349F (5ʹ‐CCCTACACGACGCTCTTCCGATCTN‐3ʹ) and 806R (5ʹ‐GACTGGAGTTCCTTGGCACCCGAGAATTCCA‐3ʹ) were used to amplify the V3–V4 regions of 16S rRNA. PCR reactions were carried out in 30 μl reaction mixtures containing 0.5 μM forward and reverse primers, 10–20 ng of template DNA, and 15 μl 2 × Taq master Mix (Takala, Dalian, China). The thermal cycling for archaea consisted of initial denaturation at 97°C for 1 min followed by 30 cycles of denaturation at 97°C for 10 s, annealing at 57°C for 15 s, and extension at 72°C for 15 s, with a final extension at 72°C for 5 min. The PCR products were purified using the AxyPrep DNA Gel Extraction Kit (Axygen, USA) and quantified. Sequencing was performed on the Miseq 2X300 platform by Sangon Biotechnology Co. Ltd. (Shanghai, China).

### 2.5 Diversity analysis and statistical analysis

The raw sequences were sorted based on the unique sample barcodes, quality control for sample sequence using Prinseq, and removed nontarget region sequences and chimeric sequences using the Mothur program (Schloss et al., 2009) and Chimeras.uchime. The sequences were clustered into operational taxonomic units (OTUs) defined by 97% similarity (3% max distance) using Uclust (Edgar, 2010). Venn diagram was charted according to the distribution of OTUs in the samples by Mothur and R language package “VennDiagram”. Alpha and beta diversity were calculated using the QIIME software package (Caporaso et al., 2010). For alpha diversity analysis, Chao1, Shannon, ACE, Coverage, and Simpson indices were calculated by Mothur program, and rarefaction curves were generated. Species were classified by RDP classifier (Wang, Garrity, Tiedje, & Cole, 2007) according to the silva database, and statistics the relative abundance at phylum, class, order, and genus level. For beta diversity analysis, cluster analysis was preceded by 2D Principal Coordinates Analysis (PCoA) using the QIIME software package. QIIME calculates both unweighted and weighted UniFrac distance (Kuroda et al., 2015; Zhang, Sun, Zeng, Chen, & Sun, 2015). Canonical correspondence analysis (CCA) was performed to discern the correlations between the archaeal community and operational parameters, including the substrate, OLR, NH_4_^+^–N, and VFA.

## 3 RESULTS AND DISCUSSION

### 3.1 Bioreactor performance

The physical and chemical parameters of the three reactors at different OLRs were shown in Table 2. These values were the average of every stage. The average specific methane yield (SMY) of R2 (PM and MS) was 218 and 254 ml g^−1^ day^−1^ at OLR of 2 and 4 g L^−1^ day^−1^, respectively, which were 7.4%, 10.4%, and 33.7%, 192.0% higher, respectively, compared with that in R1 and R3. This difference could be explained by the optimal *C*/*N* ratio of 26 in R2. In R1 (PM), the average SMY at an OLR of 4 g L^−1^ day^−1^ reached 230 ml g^−1^ day^−1^, which was 8.0% higher than that at OLR 2 g L^−1^ day^−1^. This was consistent with the report by Bolzonella, Pavan, Battistoni, and Cecchi (2005) which demonstrated that a higher OLR (up to 4 g L^−1^ day^−1^) for shorter SRTs (15 days) generated higher methane levels under both mesophilic and thermophilic conditions. However, the value decreased by 46.6% in R3 (MS), this was supported by the research of Zhou et al. (2017), who reported that biogas yield improved when OLR increased at levels below 2 g L^−1^ day^−1^ during AD of rice straw.

**Table 2:** Process parameters of the three reactors used in this study

Reactor	OLR (g L^−1^ day^−1^)	SMY^a^ (ml g^−1^ day^−1^)	NH_4_^+^‐N (mg L^−1^)	Acetic acid (mg L^−1^)	Propionic acid (mg L^−1^)	VFAs^b^ (mg L^−1^)	VFAs/Alk^c^
R1	2	203 ± 18	2,482 ± 65	85.3	15.7	141.0	0.01
	4	230 ± 20	4,384 ± 53	608.2	103.0	965.5	0.06
R2	2	218 ± 22	1,572 ± 55	100.1	24.6	149.2	0.02
	4	254 ± 10	1,020 ± 33	82.1	4.1	94.4	0.02
R3	2	163 ± 17	1,175 ± 98	49.2	27.2	108.9	0.02
	4	87 ± 8	22 ± 3	165.5	59.3	264.2	0.18

***Note***. Values are expressed as average ± *SD*.

a Specific methane yield.

b The values of VFAs were shown as acetic acid.

c Alkalinity.

Ammonia is an important nitrogen source for the growth of methanogens as well as a key pH‐stabilizing agent for the neutralization of VFAs. However, high concentration of ammonia inhibit the activity of methanogens (Li, Liu, et al., 2015). Ammonia concentration in R1 increased from 2,482–4,384 mg/L, but decreased in R2 and R3 when the OLR increased from 2 to 4 g L^−1^ day^−1^. Particularly in the case of R3, the ammonia concentration sharply decreased to 22 mg L^−1^ from 1,175 mg L^−1^. Except R1 at OLR of 4 g L^−1^ day^−1^, the ammonia concentration was lower than 3,860 mg L^−1^, which was reported as the inhibition level for methanogens (Benabdallah El Hadj, Astals, Gali, Mace, & Mata‐Alvarez, 2009). It indicated that the *C*/*N* ratio of substrate, as well as the OLR, affected the concentration of ammonia.

The concentration of VFAs is about 50–250 mg L^−1^ in a stable anaerobic digester, and the inhibitory concentration is around 1,500 mg L^−1^ (Khanal, 2008). Other studies report that AD didn't fail until up to 10,000 mg/L of acetic acid or butyric acid (Khanal, 2008), this large difference may be attributed to the buffer capacity, which maintains the pH at the desired level, as well as to the methanogens’ tolerance to toxic agents such as ammonia. In this study, the VFAs in three reactors changed when OLR was elevated: unlike the observed accumulation of VFAs in R1 and R3, acetate, propionate, and VFAs concentrations in R2 decreased by 18.0%, 83.3%, and 36.7%, respectively, as OLR increased from 2 to 4 g L^−1^ day^−1^, and propionic acid concentration was below the inhibitory threshold value of 900 mg L^−1^ (Wang et al., 2016). The ratio of propionate/acetate is also an indicator of the stability of AD process. A ratio over 1.25 was reported to induce biomethanation process failure (Wang, Wang, Cai, & Sun, 2012). In this study, the ratio was under 1.25 over the experimental period.

Although the concentration of VFAs in R1 (965.5 mg L^−1^) was more than ten times that in R2 (94.4 mg L^−1^), the system was capable of sustaining its performance since the higher alkaline capacity of manure allows higher OLR without accumulation of VFAs. Cheng and Zhong (2014) studied the effect of codigestion on biogas production from AD of cotton stalk at a feed‐to‐inoculum ratio of 4:1 and 5% TS concentration of substrates, and determined that the concentration of VFAs was 320 and 3,000 mg/L during the mono‐digestion of cotton stalk and codigestion with PM, respectively.

### 3.2 Richness and diversity analysis of OTUs

**Figure 1:**
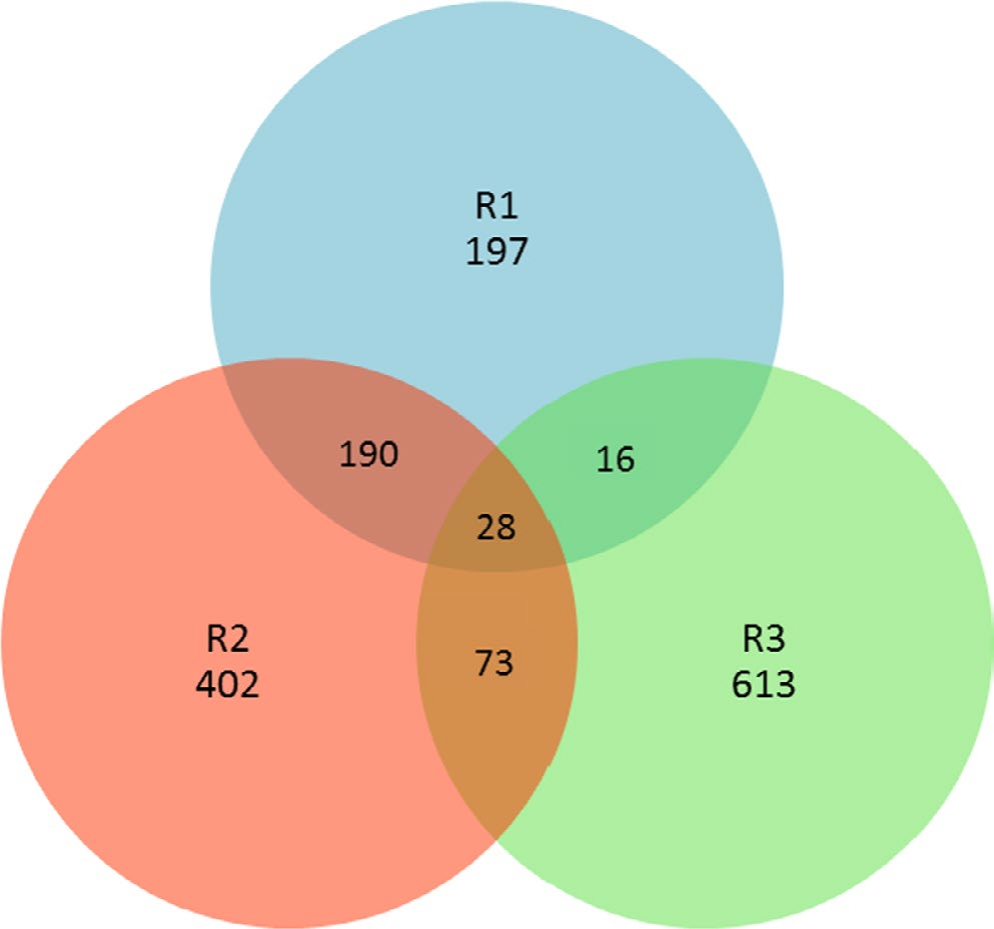
Venn diagram of the OTUs in the three samples at OLR of 4 g L^−1^ day^−1^; unique and shared OTUs among the three samples are based on 97% sequence similarity. The numbers inside the diagram indicate the number of OTUs

A total of 431, 693, and 730 OTUs from R1, R2, and R3, respectively, were obtained based on 97% sequence similarity when OLR was 4 g L^−1^ day^−1^. Venn diagram showed that R1 and R2 had maximum similarity, sharing 218 OTUs, R2 and R3 shared 101 OTUs, R1 and R3 shared 44 OTUs, and 28 OTUs were shared by the three samples (Figure 1).

**Figure 2:**
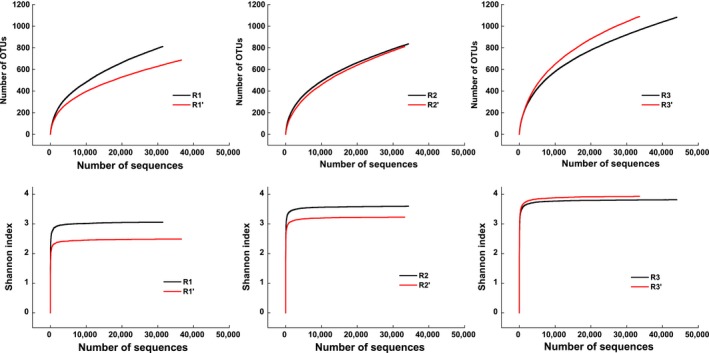
Rarefaction curves based on OTUs at 97% sequence similarity. Black and red lines represent the samples at an OLR of 2 and 4 g L^−1^ day^−1^, respectively

The diversity estimators for each sample based on a species level of 97% similarity were showed in Table 3. Rarefaction curves of OTUs profiled the change in archaeal species richness in different feedstock and OLRs (Figure 2). R3 showed the highest community diversity, followed successively by R2 and R1, as confirmed by the Simpson and Shannon index (Table 3). Although none of the rarefaction curves approached a plateau, the Shannon's diversity index rarefaction curves approached asymptotes, indicating that the sampling depths were sufficient to capture the overall microbial diversities in all six samples (Zhang, Sun, et al., 2015). The mixing of different substrates improves the nutrients in the feedstock, thereby increase the growth rate of microbial organisms, and additionally enhances digestion efficiency of the system (Li, Cheng, et al., 2016). Anaerobic codigestion of PM with MS (R2) enhances the nutritional balance and reduces the possibility of inhibition induced by lipids and ammonia. Interestingly, when the OLR was elevated, archaeal species richness decreased in R1, increased in R3, whereas that of R2 was relatively stable during the experiment.

**Table 3:** Statistical data for the archaeal community in R1, R2, and R3 at OLR of 4 g L^−1^ day^−1^

OLR	Reactor	Shannon	ACE	Chao 1	Coverage	Simpson	OTUs	Sequences
2	R1	3.06	2048	1562	0.988	0.222	812	31384
	R2	3.60	1828	1600	0.989	0.123	836	34281
	R3	3.82	2300	1980	0.990	0.088	1082	43986
4	R1	1.41	1225	877	0.994	0.568	431	36624
	R2	2.81	2412	1762	0.989	0.168	693	33225
	R3	3.56	1467	1233	0.991	0.074	730	33570

**Figure 3:**
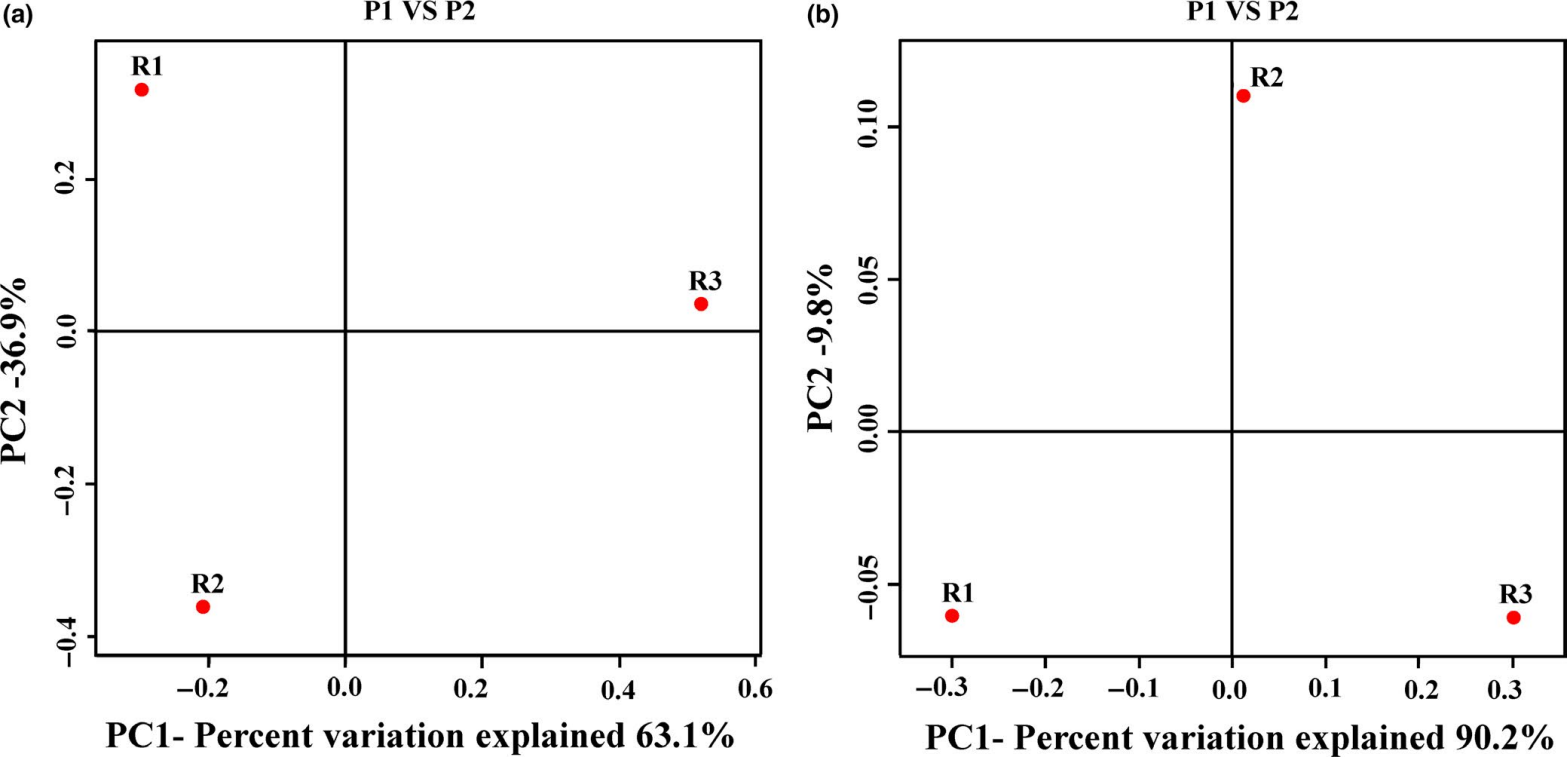
Principal Coordinate Analysis (PCoA): (a) Based on unweighted UniFrac metrics, (b) Based on weighted UniFrac metrics

To compare the methanogens community in the three samples at OLR of 4 g L^−1^ day^−1^, the principal coordinates analysis (PCoA) were performed to cluster community. Based on unweighted UniFrac PCoA, the cluster community had the maximum variation in 63.1% (PC1) and 36.9% (PC2) (Figure 3a). It demonstrated a clear regional reparation, and samples from R1 and R2 tended to cluster together, whereas R3 was clearly different from them. These clustering results suggested that R1 and R2 shared similar methanogens community, which were clearly different from those in R3. Weighted UniFrac PCoA (Figure 3b) represented that R1 and R3 were grouped together along PC2 which only accounted for 9.8% of the total variations; however, the three samples were separated from each other according to PC1, which accounted for 90.2% of the total variations.

### 3.3 The methanogenic archaeal community for different substrates

**Figure 4:**
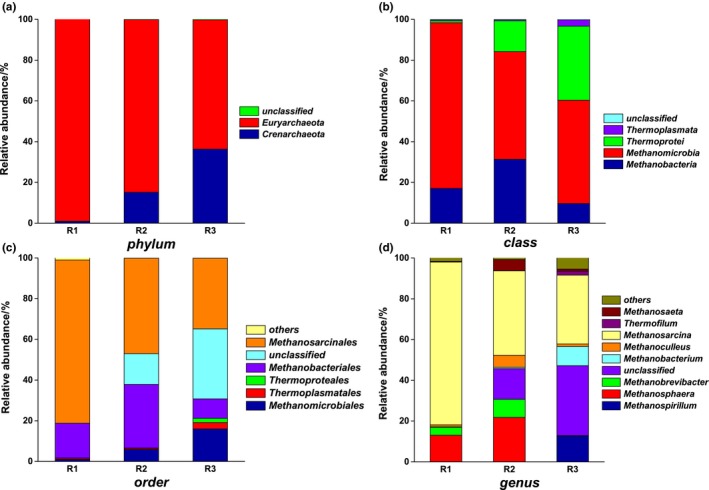
Archaeal community compositions at (a) phylum level, (b) class level (c), order level, and (d) genus level at an OLR of 4 g L^−1^ day^−1^

Archaeal community compositions of different substrates at OLR of 4 g L^−1^ day^−1^ were compared (Figure 4). The majority community detected from the three reactors was classified to the phylum *Euryarchaeota* and *Crenarchaeota*. The relative abundance of *Euryarchaeota* was 99.0%, 84.8%, and 63.5%, and that of *Crenarchaeota* was 1.0%, 15.2%, and 36.3% in R1, R2, and R3, respectively (Figure 4a). The findings indicate that maize straw affected the *Crenarchaeota* levels.

At the class level (Figure 4b), *Methanomicrobia* was predominant in R1 (81.3%), R2 (52.8%), and R3 (50.8%), respectively. *Thermoprotei* accounted for 15.2% and 36.3%, implying that no other class was affiliated with *Crenarchaeota* in R2 and R3. *Methanobacteria* in the three reactors accounted for 17.0%, 31.3%, and 9.6% of the total effective archaeal sequences, respectively.

The predominant order was *Methanosarcinales*, which accounted for 82.2%, 47.0%, and 34.8% in the three reactors. The other two dominant orders were *Methanobacteriales* and *Methanomicrobiales*;* Methanobacteriales* accounted for 31.3% of total sequences in R2, which was higher than that for R1 (17.0%) and R3 (9.6%). The relative abundance of *Methanomicrobiales* was 1.1%, 5.8%, and 16.0% in R1, R2, and R3, respectively (Figure 4c). Although *Methanobacteriales* and *Methanomicrobiales* were both hydrogenotrophic methanogens, they showed different correlation pattern with regard to AD performance; *Methanobacteriales* appeared to play an important role in high loading AD, in contrast to the negative correlation of the *Methanomicrobiales* to biogas and OLR (Vrieze et al., 2015). The SMY of R1 (230 ml g^−1^ day^−1^), R2 (254 ml g^−1^ day^−1^), and R3 (87 ml g^−1^ day^−1^) fitted the correlation pattern of *Methanobacteriales* and *Methanomicrobiales*. The relative abundance of unclassified order increased sharply with the addition of MS, from 0.1% (R1) to 15.1% (R2), and finally reached 34.4% in R3 (feeding with MS only).

The composition of methanogens at the genus level was further investigated to evaluate the influence of substrate on the archaeal community and biogas production performance (Figure 4d). Seven genera of methanogens, namely *Methanosarcina*,* Methanospirillum*,* Methanosphaera*,* Methanoculleus*,* Methanobrevibacter*,* Methanobacterium,* and *Methanosaeta*, were identified in this experiment. In the case of *Methanosarcina*, which has been often reported as the dominant methanogen in AD, the relative abundance was 79.8%, 41.5%, and 33.7% in R1, R2, and R3, respectively. This result was consistent with the finding that the most abundant sequences (41.1%) belonged to *Methanosarcina* during the anaerobic codigestion of pretreated wheat straw with cattle manure (Song & Zhang, 2015). Unlike that of *Methanosarcina*, the relative abundance of *Methanosaeta* was as low as 0.4%, 5.5%, and 1.0% in the three reactors, respectively. *Methanosphaera* was the second most abundant in R1 (13.1%) and R2 (21.8%). *Methanospirillum* and *Methanobacterium,* which were observed only in R3, were the other dominant genera, accounting for 12.6% and 9.4%, respectively.

Among methanogens, *Methanosarcina* and *Methanosaeta* are well‐known for utilizing acetate for methanogenesis. *Methanosaeta* is specialized in producing methane by acetate cleavage, whereas *Methanosarcina* is a relative generalist whose metabolic features are diverse and include both acetoclastic and hydrogenotrophic pathways (Li et al., 2013; Liu & William, 2008). Compared with *Methanosarcina*, the relative abundance of *Methanosaeta* was very low. The great difference in the relative abundances of *Methanosaeta* (0.4%) and *Methanosarcina* (79.8%) in R1 may be attributed to their tolerance to high concentrations of toxic ionic agents (ammonia concentration of 4,384 mg L^−1^) due to their ability of growing in aggregates and forming irregular cell clumps. Furthermore, high acetate concentrations favor the growth of *Methanosarcina*, which requires a minimum concentration of about 60.1 mg L^−1^ and predominates at acetate concentrations above 234.3 mg L^−1^. In contrast, *Methanosaeta* requires concentrations as low as 0.3 mg L^−1^, and not exceeding 140.6 mg L^−1^ (Liu & William, 2008). In this study, the concentration of acetic acid in the three reactors was not in the optimal concentration range for *Methanosaeta* (though not so for *Methanosarcina*) and induced selective proliferation of *Methanosarcina* (Guo, Wang, Sun, Zhu, & Wu, 2014). In addition, the intermittent stirring in completely mixed reactor may be responsible for conferring an advantage to *Methanosarcina* since *Methanosarcina* was reported to be frequently predominant in fixed and stirred tank digesters (Liu & William, 2008).

Other methanogens, such as *Methanosphaera*,* Methanospirillum*,* Methanoculleus*,* Methanobrevibacter*, and *Methanobacterium* detected in this study are hydrogenotrophic methanogens. In R1, the relative abundance of acetoclastic methanogens was 80.2%, which was much higher than that of hydrogenotrophic methanogens (total relative abundance was 18.1%); therefore, it can be concluded that acetoclastic methanogens represent a major pathway for the digestion of PM. However, for R2 and R3, the relative abundances of acetoclastic methanogens were 46.9% and 34.8%, and that of hydrogenotrophic methanogens 37.2% and 23.5%, respectively. Therefore, acetoclastic and hydrogenotrophic pathways for methanogens occur at approximately equal extents during the digestion of the PM and MS mixture, or that of MS solely. This result was consistent with the finding that the methane‐producing microbial community is involved in the anaerobic codigestion of pretreated wheat straw with cattle manure and solid state codigestion of kitchen waste, pig manure, and excess sludge (Li et al., 2013; Song & Zhang, 2015).

In addition, a large proportion of archaeal sequences belonging to class *Thermoprotei*, phylum *Crenarchaeota* were unclassified, especially in R3 (34.4%); this proportion was approximately equal to that of *Methanosarcina* (33.7%). Unfortunately, no further data on the predominance of this microorganism were available, owing to the lack of information regarding archaea in recent reports; however, this microorganism may be significant for methanogens, especially for digestion with MS as mono or multiple substrates (Francisci, Kougias, Treu, Campanaro, & Angelidaki, 2015).

### 3.4 The methanogenic archaeal community at different OLRs

**Figure 5:**
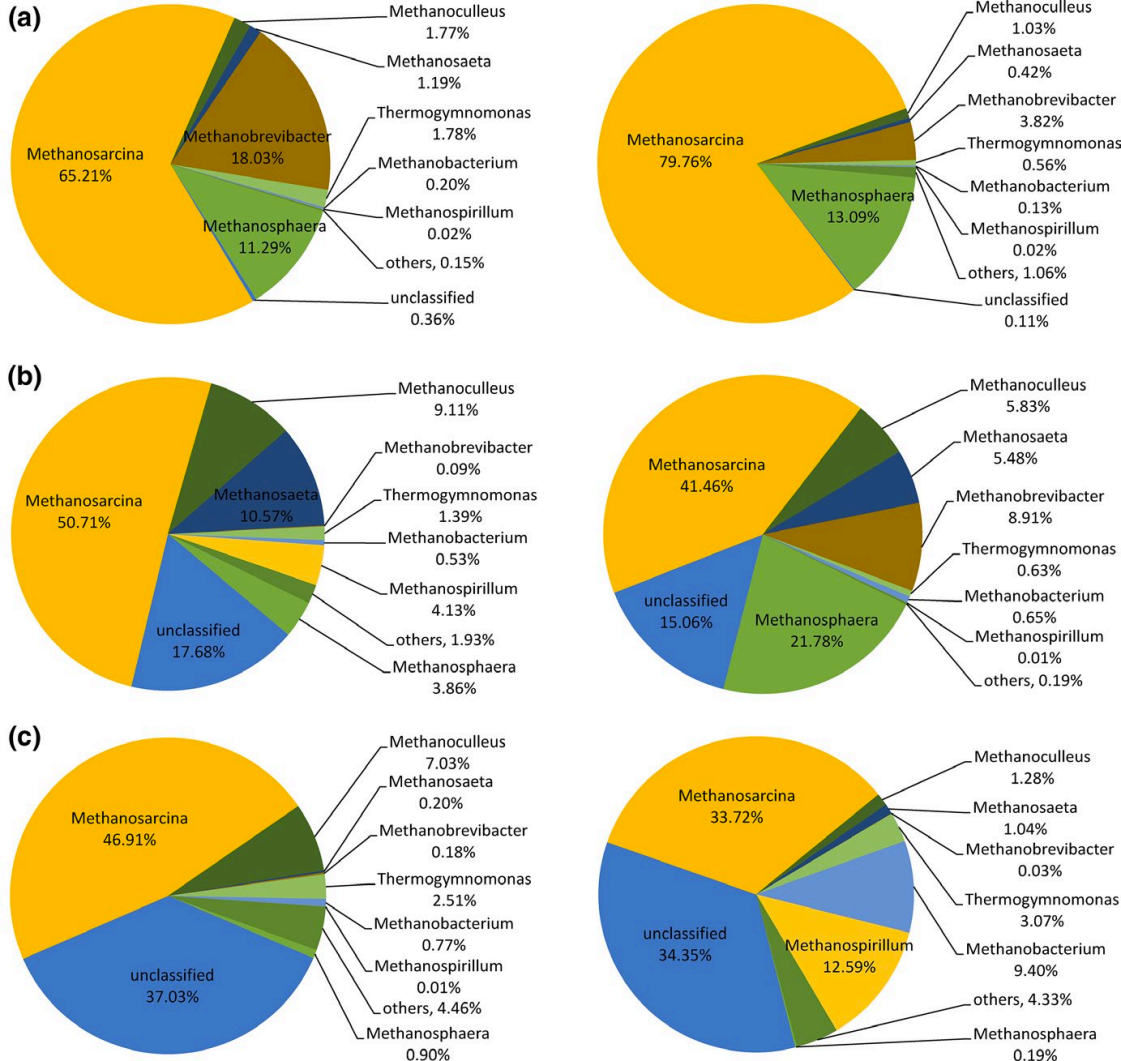
Relative abundance of methanogens 16S rDNA gene sequences of R1 (a), R2 (b), and R3 (c) at OLR of 2 g L^−1^ day^−1^ (left) and 4 g L^−1^ day^−1^ (right). The sequences showing a percentage of reads below 1.0% in all samples were grouped into ‘Others’

The genus *Methanosarcina* represented the predominant phylotype under different OLRs (Figure 5), accounting for 33.7%–79.8% of all sequencing reads in the three reactors. In R1, the main change was observed with regard to *Methanosarcina* and *Methanobrevibacter*; the relative abundance of *Methanosarcina* increased from 65.2% to 79.8%, whereas that of *Methanobrevibacter* decreased from 18.0% to 3.8%. The hydrogenotrophic methanogens accounted for 31.3% to 18.1%. In R2, although the richness of methanogens showed no obvious change (Figure 2); the archaeal community showed a clear shift when OLR increased. The relative abundances of acetoclastic methanogens genera *Methanosarcina* and *Methanosaeta* decreased from 50.7% to 10.6% to 41.5% and 5.5%, respectively, whereas that of hydrogenotrophic *Methanobrevibacter* and *Methanosphaera* increased from 0.1% to 3.9% to 8.9% and 21.8%, respectively. The relative abundance of *Methanoculleus* decreased from 9.1% to 5.8%; *Methanoculleus* has been reported to show high identity with methanogenic archaea in stable anaerobic cellulose‐degrading reactors (Chin, Lukow, Stubner, & Conrad, 1999). The total abundance of hydrogenotrophic methanogens increased to 37.2% from 17.7% when OLR was elevated. In R3, the relative abundance of *Methanosarcina* decreased from 46.9% to 33.7%, that of *Methanoculleus* decreased from 7.0% to 1.3%, and that of *Methanobacterium* increased from 0.8% to 9.4%. *Methanospirillum*, which was also present, showed a relative abundance of 12.6%. The total abundance of hydrogenotrophic methanogens increased from 8.9% to 23.5%.

*Methanosarcina w*as predominant in the three reactors, and its advantage in R1 was enhanced with the increase in ammonia concentration from 2,482 to 4,384 mg L^−1^. In previous studies (Li, Liu, et al., 2016), the stable CH_4_ production that accompanies an increase in ammonia level may be explained by the increasing activity of hydrogen‐utilizing methanogens. This is because hydrogenotrophic methanogens are capable of tolerating ammonia concentrations of 6,000 mg L^−1^, which is two folds higher than the threshold ammonia concentration for *Methanosarcina*.

In this study, although the ammonia concentration increased strongly, the relative abundance of hydrogenotrophic methanogens decreased from 31.3% to 18.1%, and the SMY increased from 303 to 330 ml g^−1^ day^−1^. Since the inoculum was cultured with high ammonia during AD feeding with PM, the ammonia in R1 (2,482–4,384 mg/L) had no inhibition for *Methanosarcina*. In addition, acetate has a crucial impact on the presence and relative abundance of acetoclastic methanogens (Yu et al., 2014); the acetate concentration in the three reactors was higher than the threshold value that favors the growth of *Methanosaeta*. With increased in OLR, the relative abundance of *Methanosarcina* decreased, whereas that of hydrogenotrophic methanogens in R2 and R3 increased by 2.1 and 2.6 folds, respectively. Furthermore, the ratio of acetoclastic/hydrogenotrophic methanogens declined to 1.26 and 1.48 from 3.46 to 5.30, which indicated that the methanogenic pathway apparently shifted from mainly acetoclastic to the coexistence of acetoclastic and hydrogenotrophic methanogens pathways in the system (Goux et al., 2015). The coexistence of methanogenic pathways seems necessary for response to perturbation and maintenance of stable process performance (Lerm et al., 2012); this was evidenced by the performance of R2, in which the SMY increased by 13.3%, whereas it decreased by 46.6% in R3.

### 3.5 The correlation between archaeal population and reactor parameters

**Figure 6:**
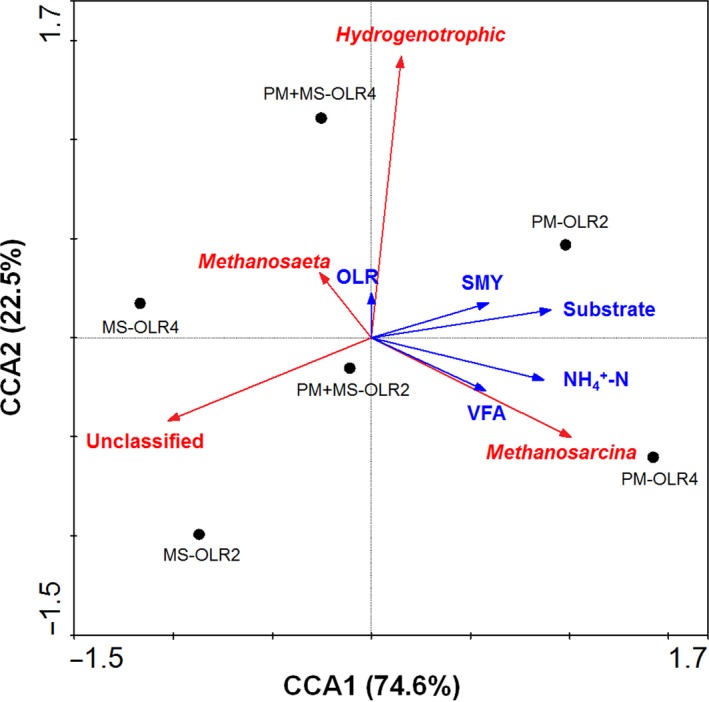
Canonical correspondence analysis (CCA) of the archaeal community and the operational parameters in the three reactors. Blue vectors represent the influence of the process parameters such as OLR, SMY, substrate type, VFA, and NH_4_^+^‐N; red vectors represent methanogenic archaea identified by high‐throughput sequencing at the genus level. Black points represent the substrate type at different OLRs

Canonical correspondence analysis (CCA) was used to highlight the influence of altered process parameters on the archaeal community (Goux et al., 2015). As shown in Figure 6, the first and second canonical axes represented 74.6% and 22.5% of variance, respectively. Compared with the OLR, substrate type and ammonia were the biggest influencing factors for the study probably because pig manure contains high amounts of protein. The archaeal community was segregated by substrate, and OLR indicated that substrate type and loading could segregate archaeal community in these anaerobic digesters. OLR2 was distinguished by the second canonical axis from OLR4 in all reactors, and R1 was separated from the other reactors by the first axis; these distinctions suggest that a significant shift in the archaeal community occurred in all reactors (Jang, Kim, Ha, & Park, 2014). These results agree with those for the taxonomic distribution of archaeal community (Figure 5).

CCA revealed that substrate, ammonia, and VFA play key roles in determining the community structure; these factors are positively correlated with the abundance of *Methanosarcina*. On the contrary, the presence of *Methanosaeta* was negatively correlated with these factors, and with the abundance of *Methanosarcina*. These findings may be attributed to the greater tolerance of *Methanosarcina* for ammonia and VFA relative to that of *Methanosaeta* (Hao et al., 2016). The analysis also showed a clear positive relationship between OLR and hydrogenotrophic methanogens; with an increase in OLR, the relative abundance of hydrogenotrophic methanogens increased, whereas that of *Methanosarcina* decreased in R2 and R3, and the concentrations of ammonia and VFA decreased (Table 2, Figure 5).

## 4 CONCLUSIONS

High‐throughput sequencing data showed differences in archaeal community; *Euryarchaeota* and *Crenarchaeota* constituted the majority community; the relative abundances were approximately 63.5%–99.0% and 1.0%–36.3%, respectively. *Methanosarcina*, which accounted for 33.7%–79.8%, represented the predominant genus. The richness of archaeal community during codigestion of pig manure with maize straw (ACE: 2412) was greater, the diversity during the digestion of maize straw was higher (Shannon: 3.56). With increase in OLR, methanogenic archaea showed larger shifts in all reactors. A shift from acetoclastic (*Methanosarcina*) to hydrogenotrophic methanogens was observed in the reactors of the mixture or maize straw only; VFA, but not high ammonia concentration, could probably be the reason. Further studies should focus on the unclassified genus during digestion of maize straw.

## CONFLICT OF INTEREST

The authors declare no conflict of interest.

## Data Availability

All data generated or analyzed during this study are included in this published article.
